# The Architecture of Risk for Type 2 Diabetes: Understanding Asia in the Context of Global Findings

**DOI:** 10.1155/2014/593982

**Published:** 2014-03-13

**Authors:** Noraidatulakma Abdullah, John Attia, Christopher Oldmeadow, Rodney J. Scott, Elizabeth G. Holliday

**Affiliations:** ^1^School of Biomedical Sciences and Pharmacy, Faculty of Health, University of Newcastle, Newcastle, NSW 2308, Australia; ^2^UKM Medical Molecular Biology Institute (UMBI), Universiti Kebangsaan Malaysia, Kuala Lumpur, Malaysia; ^3^Clinical Research Design, IT and Statistical Support (CReDITSS) Unit, Hunter Medical Research Institute, Newcastle, NSW 2305, Australia; ^4^Centre for Clinical Epidemiology and Biostatistics, School of Medicine and Public Health, Faculty of Health, University of Newcastle, Newcastle, NSW 2305, Australia; ^5^Hunter Area Pathology Service, John Hunter Hospital, Newcastle, NSW 2305, Australia

## Abstract

The prevalence of Type 2 diabetes is rising rapidly in both developed and developing countries. Asia is developing as the epicentre of the escalating pandemic, reflecting rapid transitions in demography, migration, diet, and lifestyle patterns. The effective management of Type 2 diabetes in Asia may be complicated by differences in prevalence, risk factor profiles, genetic risk allele frequencies, and gene-environment interactions between different Asian countries, and between Asian and other continental populations. To reduce the worldwide burden of T2D, it will be important to understand the architecture of T2D susceptibility both within and between populations. This review will provide an overview of known genetic and nongenetic risk factors for T2D, placing the results from Asian studies in the context of broader global research. Given recent evidence from large-scale genetic studies of T2D, we place special emphasis on emerging knowledge about the genetic architecture of T2D and the potential contribution of genetic effects to population differences in risk.

## 1. Introduction

Type 2 diabetes (T2D) is one of the top five noncommunicable diseases globally, comprising a major, growing cause of morbidity and premature death. In 2012, the International Diabetes Federation (IDF) estimated that 371 million people worldwide were living with diabetes, of which about half live in South Asia, the Western Pacific, and Eastern Mediterranean regions [1]. Asia is now the epicenter of an escalating diabetes epidemic, chiefly due to population growth and ageing in India and China. Projections suggest that by 2030, more than 60% of worldwide diabetes cases will come from Asia [2, 3], with the vast majority of these being Type 2 diabetes (T2D) [4]. T2D has an enormous economic, psychosocial, and physical impact on individuals, their families, and communities, both directly and indirectly. The direct economic burden of T2D includes both recorded expenditure by health services and unrecorded costs borne by patients and their families. Indirect costs such as loss of productivity and disability are also substantial and may match or surpass direct costs. The proportion of worldwide disability-adjusted life years (DALYs) due to T2D has soared in recent decades, rising from 43% in 1990 to 54% in 2010 [5]. Temporary and permanent disability, excess morbidity, and premature death are the consequences of T2D vascular complications, including cardiovascular disease, retinopathy (blindness), nephropathy (kidney failure), and neuropathy (nerve problems) which can lead to amputation. Intangible costs due to psychosocial effects on quality of life, diminished contribution to family tasks, and reduced income of care-giving family members are also likely substantial but difficult to assess. The enormous, growing global burden of T2D—particularly in Asia—is now viewed as a crisis by the World Health Organisation (WHO) and the United Nations (UN) [6]. There is a major worldwide push to decrease the prevalence and impact of T2D by identifying risk factors, both genetic and nongenetic. Explaining the distribution and variation of T2D susceptibility across Asia will be vital for reducing the global burden of disease, due to the demographic, cultural, and genetic heterogeneity of Asian populations, and T2D risk factor profiles between these populations [7–10].

## 2. Epidemiology

### 2.1. Burden of the Disease

The vast majority of T2D (about 80%) occurs in low- and middle-income countries (LMICs), with India and China providing the largest absolute contributions. The prevalence of T2D is also rising most swiftly in LMICs [6], particularly in Asian countries experiencing rapid economic growth (Figure 2). However, there are disparities in T2D prevalence among Asian populations; Asians from the Indian subcontinent (India, Pakistan and Bangladesh) have the highest prevalence (15.9% to 24.9%), with intermediate prevalence in Malays (11.4% to 16.9%) and reduced prevalence in Chinese (6.4% to 13.8%) [11–13]. These risk profile differences may reflect population differences in T2D risk due to ethnicity-specific diet and lifestyle, body composition, genetic effects, or gene-environment interactions, as discussed further in the sections below.

### 2.2. Pathophysiology

The pathogenesis of Type 2 diabetes (T2D) involves deficient insulin secretion by pancreatic *β*-cells, and diminished insulin effectiveness in target tissues (insulin resistance) T2D aetiology differs from that of Type 1 diabetes (T1D), in which there is absolute insulin deficiency due to the destruction of insulin-producing *β*-cells [14]. T2D represents 90% of all diabetes cases worldwide [4]. Impaired insulin secretion and insulin action led to an accumulation of glucose in the blood (hyperglycaemia), with adverse effects on health. Clinical features of hyperglycaemia and T2D include excessive excretion of urine (polyuria), thirst (polydipsia), constant hunger, weight loss, vision change, and fatigue [15]. These symptoms may occur suddenly but are often less marked, and T2D patients may be unaware of their illness for several years until further complications develop.

#### 2.2.1. Insulin Resistance

Glucose homeostasis depends upon a highly regulated feedback system comprising both insulin-secreting *β*-cells and insulin-sensitive target tissues. The function of either component—while accounting for the associated homeostatic response of the other—can be evaluated using Homeostasis Model Assessment (HOMA) [16]. Studies assessing insulin resistance using HOMA (HOMA-IR) report continental differences in the relative contribution of insulin deficiency versus insulin resistance to T2D. Compared to healthy European-ancestry participants matched for age and body mass index (BMI), Asian Indian individuals exhibit higher insulin resistance [17] and a greater contribution of insulin resistance—relative to insulin secretion—to T2D pathogenesis [18]. One study evaluating insulin response to a fixed glucose load also showed that Japanese-Americans displayed an insulin response more similar to native Japanese than European-Americans, in spite of sharing a highly Westernized lifestyle with their European-American counterparts [8].

There is also variation in the predisposition to insulin resistance* between* Asian populations [19]. For several decades, it has been recognised that the highest propensity is present in Asian Indians, in whom insulin resistance contributes substantially to T2D pathogenesis [20], potentially reflecting ancestry-related predisposition to abdominal obesity [21, 22]. A recent population based study of 4,136 Chinese, Malays, and Asian Indians living in Singapore supported these findings, reporting substantially higher insulin resistance in Asian Indians, intermediate levels in Malays, and the lowest levels in Chinese (*P* < 0.001) [19]. Differences between Malays and Chinese were removed after adjusting for body mass index (BMI); the remaining additional resistance in Indians appeared to be mediated by a tendency to higher BMI and BMI-adjusted waist circumference, together with other unexplained factors [19].

Dickinson and colleagues studied postprandial hyperglycemia and insulin sensitivity after a 75 gram carbohydrate challenge in 60 lean, healthy individuals from five ethnic groups with similar age, BMI, waist circumference, and birth weight distributions. Prior to carbohydrate consumption, fasting insulin was significantly higher in South Indians and South East Asians, compared to European Caucasian, Arabic, and Chinese individuals (*P* < 0.001) [23]. Following the challenge, hyperglycemia was significantly higher in South East Asian and Chinese participants compared with European Caucasians, while Indians and South East Asians showed a 2-3-fold higher insulin response than Europeans [23]. A small Singapore-based study of 30 individuals also showed significantly reduced insulin sensitivity in South Indians compared with Chinese or European individuals matched for age, BMI, and physical activity [24].

#### 2.2.2. Insulin Secretion

Impaired insulin secretion is associated with *β*-cell dysfunction that results in a reduced insulin-secretion response to rises in blood glucose after eating [25]. The insulin secretion response to various foods can be quantified using the insulin index; more complex relationship between insulin secretion and insulin sensitivity can be measured using the disposition index (DI), which is assessed by an intravenous glucose tolerance-test [26]. A recent family-based study found that a high-fat, low-carbohydrate dietary pattern contributed to obesity, insulin resistance, and reduced *β*-cell function [27]. This finding might be explained by increased free fatty acids (FFAs) reducing the expression of *β*-cell—specific transcription factors and impairing the *β*-cells' ability to respond to glucose with appropriate insulin secretion [28].

Similar to insulin resistance, insulin secretion also shows evidence of racial differences, being reduced in Asians compared with Europeans. The insulin index of Asians is reduced almost 70% in the progression from impaired glucose tolerance (IGT) to T2D, whereas in Europeans the corresponding reduction is only 50% [29, 30]. A population based-cohort study of insulin resistance and *β*-cell function during pregnancy also found a significantly lower *β*-cell secretory response to pregnancy-induced insulin resistance in South Asian and East Asian women, compared to European participants with a similar level of insulin resistance [31].

#### 2.2.3. Complications

T2D complications can be life-threatening and include cardiovascular disease, nephropathy (kidney disease), retinopathy (blindness), and neuropathy (nerve impairment). Observational studies in European American and African American population report that cardiovascular disease risk in individuals with T2D is more than double the rate in the general population [32] and 50% of people with T2D die from cardiovascular disease, primarily heart disease and stroke [33].

There is evidence for population differences in the rate of T2D complications, between Asian populations and broader continental groups. A cross-sectional study of 5,707 Chinese, Indians, and Malays showed that the population attributable risk of ischaemic heart disease related to T2D was the highest in Indians (40.9%), intermediate in Malays (27.9%), and the lowest in Chinese (11%) [34]. A cohort study found that the progression of kidney dysfunction in T2D was faster in Indo-Asian (Indian, Pakistani, and Bangladeshi) subjects - with an estimated 2-3-fold increase in the mean rate of rise of serum creatinine over a constant follow-up period—compared to European-ancestry subjects [35]. The prevalence of diabetic end stage renal disease (ESRD) has also been reported as significantly higher in Asian T2D subjects (52.6%) compared to Caucasians (36.2%) [36].

Another microvascular complication of T2D, diabetic retinopathy, represents about five percent of all cases of global blindness [37]. Visual impairment occurs as a result of long-term, accumulated damage to small blood vessels in the retina. A recent cross-sectional study conducted by The Diabetic Retinopathy in Various Ethnic groups in UK (DRIVE UK) found that South Asian T2D populations have significantly higher prevalence of diabetic retinopathy (42.3% versus 38%) and sight threatening diabetic retinopathy (10.3% versus 5.5%) compared to white Europeans [38].

Combined with reduced blood flow, neuropathy (nerve damage) in the feet increases the risk of foot ulcers, infections, poor wound healing, and poor distal circulation, eventually increasing the risk of limb amputation [39]. Due to the elevated risk of these life-threatening complications, mortality risk among people with diabetes is at least double that of individuals without diabetes [40].

### 2.3. Conventional Risk Factors

**Figure 1:**
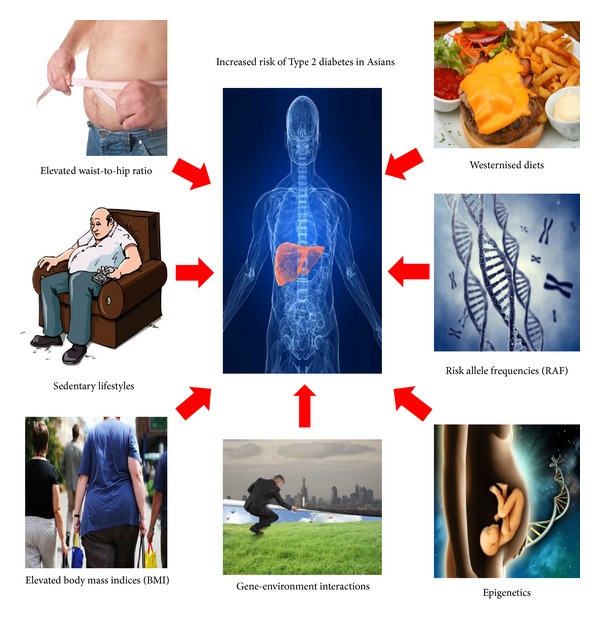
Genetic and nongenetic risk factors contributing to increased Type 2 diabetes risks within Asian populations and risk differences between Asian groups.

**Figure 2:**
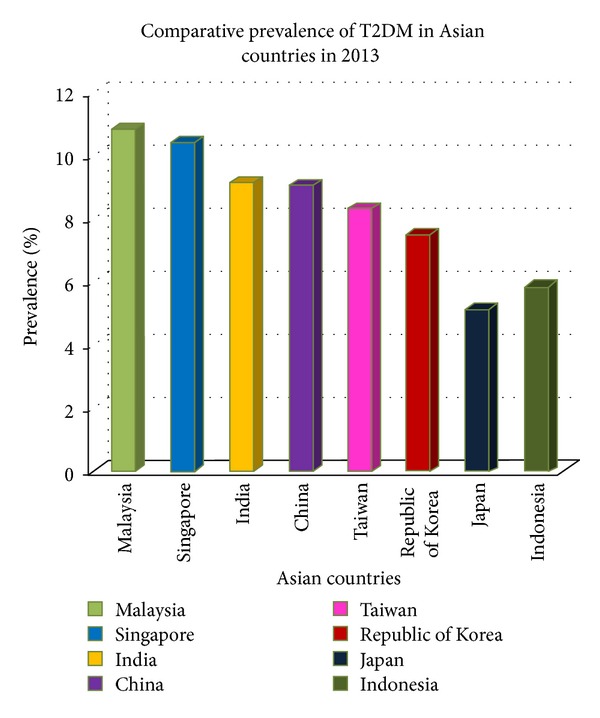
Comparative prevalence of Type 2 diabetes mellitus in Asian countries in 2013 (data source: http://www.idf.org/diabetesatlas/data-visualisations).

A range of lifestyle and clinical factors contribute to risk of insulin resistance and T2D, including elevated body mass index (BMI), high waist-to-hip ratio (WHR), physical inactivity, and diet (Figure 1).

#### 2.3.1. Body Mass Index (BMI) and Obesity

According to the World Health Organisation (WHO), body mass index (BMI) is a simple index of weight-for-height that can be widely used to classify overweight and obesity in adults [41]. It is defined as a person's weight in kilograms divided by the square of their height in meters (kg/m^2^). Individuals with BMI greater than or equal to 30 kg/m^2^ are classified as obese for international standardised comparison. Obesity elevates serum fatty acid concentrations, reducing glucose uptake and increasing fatty acid uptake by the liver, skeletal muscle, and pancreatic *β*-cells. Reduced glucose uptake elevates serum glucose, stimulating further insulin secretion; it is the lack of response to this secreted insulin that induces insulin resistance [42]. Continually high insulin secretion in turn produces metabolic stress in pancreatic *β*-cell mitochondria, inducing the release of reactive oxygen species that damage mitochondria. Over time, mitochondria lose their ability to maintain cellular processes and *β*-cells undergo apoptosis, irreversibly reducing insulin secretion potential [43].

Associations between BMI, percentage of body fat, and body fat distribution differ across populations, influencing the thresholds at which T2D risk increases. Asian T2D patients have lower average BMI compared to European patients [44], which might reflect higher percentage body fat in Asians (3–5% higher) than Europeans for a given BMI [45, 46]. Similarly, for a fixed body fat percentage, Asians have a 3 to 4 unit lower BMI than Europeans [45]. The body fat percentage is also different* between* Asian groups; for fixed BMI, it tends to be the highest in Indians, followed by Malays and Chinese [47]. One study also showed that among Asians, Indians have the highest prevalence of obesity (35.8% (95% CI: 32.4–39.3)), followed by Malays (32.0% (95% CI: 30.6–33.4)) and Chinese (19.7 (95% CI: 17.9–21.6)) [13]. However, due to differences in body composition, recent studies have shown that waist circumference (WC) measurement or waist-to-hip ratio (WHR) is a better predictor of T2D in Asian populations than simple BMI or body fat percentage [48, 49], since these latter measures are insensitive to differences in body fat distribution.

#### 2.3.2. Abdominal Obesity (High Waist-to-Hip Ratio/High Waist Circumference)

High waist-to-hip ratio (WHR) and waist circumference (WC), or abdominal obesity, is a major cause of insulin resistance since subcutaneous abdominal adipocytes drain their lipolytic products (free fatty acids) directly into the portal vein [50]. These free fatty acids are thought to decrease hepatic clearance of insulin and worsen systemic hyperinsulinemia [51], a precursor to T2D. Additional factors such as reduced secretion of adiponectin by adipose tissue may also contribute to the insulin-resistant state in individuals with abdominal obesity [52]. Adiponectin is an adipose tissue-specific protein that controls a number of metabolic processes, including insulin sensitivity and fatty acid oxidation [53].

The prevalence of abdominal obesity differs between ancestral groups and seems particularly marked in certain ethnic populations such as Native Americans, African-Americans, Asians, and Pacific Islanders [54–56]. The Multi-Ethnicity Study of Atherosclerosis found that for a given waist circumference, Chinese have the highest diabetes incidence, followed by Hispanic, African, and European-ancestry individuals [57], a finding that may be explained by higher levels of visceral adipose tissue (VAT) in Chinese compared with Europeans, at a fixed waist circumference [58]. The same study also found that South Asians have substantially higher visceral adipose tissue compared to Europeans for given waist circumference [58]. This might explain increased lipid and insulin levels observed in South Asians compared with Europeans at the same WC and/or WHR [59]. Such differences are apparent not only between Asian and other continental populations, but also among Asian populations. Among three major Asian groups, the prevalence of abdominal obesity seems to be significantly higher among Indians (61.8% (95% CI: 58.3–65.2)) compared with Malays (45.3% (95% CI: 43.8–46.8) or Chinese (40.4% (95% CI: 38.0–42.7)) [13].

#### 2.3.3. Diet and Physical Activity

The increasing global prevalence of T2D parallels escalating obesity rates resulting from reduced physical activity, increased intake of total calories, saturated fat (especially in fast food), and sugar-sweetened beverages (SSBs) in many societies. Asian populations are undergoing a nutrition transition in conjunction with the increasing adoption of Westernized lifestyles. In India and China, for example, caloric intake from animal fat has almost doubled in recent decades [60, 61]. High consumption of red and processed meat, SSBs, and refined grains with associated low consumption of cruciferous and yellow vegetables is strongly associated with increased in T2D [62]. At the same time, physical activity has reduced in Asian populations due to rapid urbanization and modernization [63, 64], further increasing T2D risk.

#### 2.3.4. Metabolic Features

Metabolic features including elevated blood pressure, hyperglycaemia, and hyperlipidaemia increase T2D risk by several-fold [65]. A recent multi-ethnic population-based survey indicated population differences in the prevalence of metabolic syndrome features, irrespective of T2D status. Indians seem to have higher levels of triglycerides and hyperglycaemia and lower HDL cholesterol, compared with Malay and Chinese [66]. These findings parallel those of a case-control study in which Indians from UK and Indians from India had higher total insulin and triglycerides and lower HDL cholesterol compared to European individuals, irrespective of shared environmental influences [22].

#### 2.3.5. Other Factors

Other factors that have been associated with T2D risk include short sleep duration [67, 68], increasing age, which may reflect reduced exercise and muscle mass [14], history of gestational diabetes, polycystic ovary syndrome, severe mental illness, and having a family history of the disease [54]. A recent randomized, crossover study found that sleep deprivation impairs peripheral metabolic pathways, thereby reducing insulin sensitivity [69]. The loss of skeletal muscle mass with age, or sarcopenia, is also related to insulin resistance, with sarcopenia thought to cause insulin resistance and thereby increase risk of diabetes [70]. In turn, insulin resistance results in further loss of muscle strength [71]. Finally, patients with severe mental illness such as schizophrenia or bipolar disorder have 3-fold higher risk of developing T2D compared to the general population; this may result from underlying lifestyle factors, adverse effects of pharmacotherapy, and possible common genetic and/or environmentally linked pathophysiologic processes [72].

### 2.4. Genetic Susceptibility

In addition to conventional risk factors, family, twin and genetic studies show that T2D susceptibility has a substantial genetic component [73]. Full siblings of T2D probands have a 30–60% increased risk of disease, compared with the general population [74, 75] and children with one affected parent have a 40% lifetime risk of developing T2D, which rises to almost 70% if both parents are affected [76]. Twin studies also show higher T2D concordance in monozygotic (60–70%) compared with dizygotic twins (20–30%) [77–79].

The proportion of trait variance due to additive genetic effects is termed “heritability” and can be formally estimated from twin studies. Twin study heritability estimates are on the order of 30–70% for T2D and about 60% for impaired glucose tolerance (IGT) [80, 81]. Twin studies also demonstrate a substantial genetic component for quantitative phenotypes related to glucose homeostasis, with heritability estimates of 75–85% for* in vivo* insulin secretion, ~50% for peripheral insulin sensitivity, and ~50% for glucose metabolism [82].

Population differences in T2D pathophysiologic and risk factor profiles have been discussed in previous sections. It has been suggested that such differences may partly reflect population differences in the frequency of particular genetic risk factors and/or population-specific interactions between genetic and environmental factors [83].

### 2.5. Methods of Gene Identification for Common Complex Disease

Observed patterns of T2D inheritance, combined with the results of recent large-scale genetic studies, suggest that the genetic component of T2D is complex, involving multiple genetic variants of individually small effect (polygenic model) [84]. There have been three main approaches employed to identify genetic risk variants for such common complex disorders: linkage studies, candidate gene association studies (CGAS), and, more recently, genome-wide association studies (GWAS).

#### 2.5.1. Linkage Studies

Familial linkage studies seek to identify broad genomic regions harboring disease risk variants by tracking disease and genetic marker segregation through multiple generations of families. Familial linkage studies are challenging for disorders with advanced age at onset, as parents may no longer be alive. Further challenges include difficulty in collecting accurate genealogical information and genetic (locus) heterogeneity, meaning that a particular risk locus contributes to disease in only a subset of families [85]. More broadly, this approach is limited by low power for common variants of small effect [86] and its inability to precisely localise underlying risk variants [87]. Earlier linkage studies found four (4) genetic loci linked with T2D;* CAPN10* [88],* ENPP1* [89],* HNF4A* [90], and* ACDC* (*ADIPOQ*) [91]. However, only the* HNF4A* locus has been confirmed by recent large-scale genome-wide association studies (GWAS) [92].

*HNF4A*, together with the related locus,* HNF1A* and also* GCK* also account for up to 80% of rare monogenic forms of diabetes. These diabetes cases present as familial, young onset, noninsulin dependent diabetes mellitus (maturity onset diabetes of the young or MODY) and are inherited in a Mendelian dominant pattern [75]. Unlike common polygenic forms of T2D, these monogenic forms require only one pathogenic genetic variant to produce disease.

#### 2.5.2. Candidate Gene Association Studies (CGAS)

In contrast to linkage studies, the candidate gene approach searches for associations between common genetic variants and disease, restricting the search region to prespecified genes of interest. Candidate genes are typically selected based on* a priori* knowledge or hypotheses reflecting the gene's presumed biological role in disease [93]. The most common study design is the case control study; for a particular genetic variant, this involves comparing the frequency of genetic alleles between individuals with and without disease, aiming to identify alleles that are associated with disease status [87]. Although a mainstay of the initial era of disease gene mapping, the candidate gene approach has been limited by small sample sizes, restriction of hypotheses to known biology, and an inability to replicate many results [94]. While candidate gene studies have reported numerous variants as beeing associated with T2D [75], just three loci,* PPARG* [95],* KCNJ11* [96], and* TCF7L2* [97], have been robustly confirmed by recent GWAS [74, 98, 99]. We note that the* TCF7L2 *association study was informed by prior genome-wide linkage study showing suggestive linkage between T2D and the 10q genomic region harboring* TCF7L2 *[97]. 

#### 2.5.3. Genome-Wide Association Studies (GWAS)

Within the last five years, genome-wide association studies (GWAS) have emerged as the method of choice for identifying common genetic variants associated with complex disease. GWAS were facilitated by completion of the Human Genome Project in 2003, the International HapMap Project in 2005 that catalogued millions of common single nucleotide polymorphisms (SNPs), and the parallel development of high throughput genotyping arrays. Single nucleotide polymorphisms (SNPs) are DNA sequence variants in which a single nucleotide differs between individuals. SNPs have a low historical mutation rate, are amenable to high throughput genotyping, and are distributed abundantly across all 22 autosomes and the sex chromosomes. Typically several million variants are screened genome-wide; appropriate adjustment of the prespecified significance level is thus necessary to avoid an increase in false positive results. Based on patterns of human genomic correlation, Bonferroni correction for one million independent tests is the accepted approach, with variants required to reach a pointwise *P* value < 5 × 10^−8^ (or 0.05/1,000,000) to be declared “genome-wide significant” [100]. Due to this stringent significance level, very large sample sizes are necessary to identify associations of modest effect, which is often achieved via international collaboration and the formation of consortia. The existence of such collaborations also facilitates rapid replication of findings in independent samples, a requirement for publication.

It has been eight years since the first notable GWAS finding in 2005, identifying a common allele of large effect associated with age-related macular degeneration. The year 2007 was coined the “Year of GWAS”, due to the explosion of GWAS publications in that year. From 2005 to September 2013, there have been more than 1,600 GWAS published reports for a range of human diseases and traits, with an online catalogue established by the National Human Genome Research Institute at the US National Institutes of Health to collate major findings (http://www.genome.gov/gwastudies/). Although complicated and costly, GWAS have successfully identified thousands of genetic loci associated with common complex diseases under the common disease common variant (CDCV) hypothesis.

### 2.6. Genome-Wide Association Studies of T2D

The first T2D GWAS was published in 2007 [99], and as of September 2013, there were more than 40 publications on T2D and its complications listed in the online catalogue of published genome-wide association studies (http://www.genome.gov/gwastudies/). At the time of writing, the catalogue describes over 100 individual SNPs showing genome-wide significant association (*P* < 5 × 10^−8^) with T2D and related metabolic traits across diverse populations (Table 1) and over 60 SNPs showing suggestive association (*P* < 1 × 10^−5^) (Table 2). This section will provide a review of T2D GWAS findings to date.

The first T2D GWAS was conducted in European-ancestry participants 2007 by Sladek and colleagues [99], using a discovery sample of 600 cases and 600 controls and a separate European replication sample of nearly 3,000 cases and 3,000 controls. This small study of early onset T2D reported T2D-associated variants in three novel susceptibility genes:* TCF7L2 *and* HHEX/IDE* which are associated with *β*-cell function [101] and* SLC30A8*, encoding a zinc transporter highly expressed in pancreatic islets [102]. Several months later, four additional European studies [74, 98, 103, 104] confirmed association of variants at these loci and identified additional associated variants in* IGF2BP2*, associated with *β*-cell dysfunction [105], and* CDKN2A/CDKN2B* and* CDKAL1*, which are both associated with *β*-cell development [105, 106]. During this time, variants in the* FTO* (fat mass and obesity associated) gene were also identified with important effects on obesity and hence, indirectly, T2D [107, 108]. Interestingly, as the effect of* FTO* variants on T2D is only via obesity, the* FTO* locus was not identified in T2D GWAS using cases and controls matched for BMI. Two of these early publications also showcased the output of international consortia: The UK-based Wellcome Trust Case Control Consortium (WTCCC) and the USA-based Diabetes Genetics Initiative (DGI), highlighting the benefits of large-scale collaboration in the GWAS era.

Since these initial GWAS had modest power to detect variants with modest effects on disease risk, follow-up studies employed meta-analysis to increase sample size and hence power to detect additional loci of similar or smaller effect. The first T2D GWAS meta-analysis was published in 2008 and was also a European study [109], representing collaboration between three different consortia; the WTCCC, DGI, and the Finland—United States Investigation of NIDDM Genetics (FUSION) which combined to form the Diabetes Genetics Replication and Meta-Analysis (DIAGRAM) consortium. This study utilised an enlarged discovery sample of 4,549 cases and 5,579 controls with replication in further 24,194 cases and 55,598 controls, all of European-ancestry. This study identified associated variants at six additional novel loci:* JAZF1, CDC123, TSPAN8 *and* THADA *which are associated with *β*-cell dysfunction [110, 111],* ADAMTS9 *which is associated with insulin action [111], and* NOTCH2*, associated with glucose-stimulated glucagon secretion by pancreatic islet cells [112].

These initial T2D GWAS were all restricted to populations of European-ancestry. The first two large-scale T2D GWAS conducted in Asian populations were reported in 2008, each employing a multi-stage design in East Asian groups. Both studies reported association of variants in* KCNQ1*, encoding the alpha subunit of a voltage-gated potassium channel expressed in the pancreas [113, 114]. These studies clearly demonstrated the utility of extending T2D GWAS to non-European populations; association of the* KCNQ1* variants with T2D was not detected in previous European GWAS, due to markedly lower frequency of the risk allele in Europeans (5% versus 40%), resulting in dramatically reduced power. A European meta-analysis subsequently confirmed association of the* KCNQ1* variants with T2D in Europeans but at significance levels far below thresholds usually inspiring replication or follow-up studies (*P* ~ 0.02).

A European study published in 2009 used multiple samples of French, Danish, and Finnish ancestry to identify association of variants in the insulin receptor substrate 1 gene (*IRS1*), showing that the risk allele is also associated with insulin resistance and hyperinsulinaemia in large population-based cohorts [115]. This contrasted with the apparent biology of previous associations, which principally related to impaired pancreatic *β*-cell function.

This first wave of T2D GWAS was succeeded by a second wave beginning in 2010, in which existing and new datasets were combined into expanded meta-analyses. The most notable was a large European study reported by Voight and colleagues, involving ~42,000 T2D cases and 100,000 controls split between discovery and replication stages and identifying twelve new associated loci. These included X-chromosomal association and an* HNFA1A* locus overlapping with the locus implicated in Mendelian monogenic (single gene) forms of diabetes [116]. Other studies reported at this time included three East Asian studies, one African American, and one European study, which together identified nine (9) additional loci [117–121]. Three of these genes have unknown function (*RBMS1, PTPRD*, and* SRR*) [117, 118], while* RPS12, LIMK2*, and* AUH* are associated with diabetic nephropathy [121],* C2CD4A* is associated with *β*-cell dysfunction [119, 122],* SPRY2* is associated with obesity and insulin resistance [120, 123], and* SASH1* is associated with insulin growth factors [121].

A subsequent 2011 meta-analysis included three Southeast Asian populations: Chinese (3955 subjects), Indian (2146 subjects), and Malay (2034 subjects) and it further emphasized the value of surveying diverse ethnic groups [124]. This study was the first to include individuals from the Malay population, the largest group in Southeast Asia, with a population size of more than 300 million [124]. This study alone contributed an additional 16 novel loci, in spite of its relatively modest sample size; this partly reflected higher minor allele frequencies in Southeast Asian populations at some associated loci (e.g., rs3792615, number 18 in Table 2).

The first T2D GWAS in South Asian populations was also published in 2011, including individuals from India, Pakistan, Sri Lanka and Bangladesh. Using a relatively modest sample size (5,561 cases and 14,458 controls in the discovery step) five additional novel T2D loci were discovered [92]: HNF4A, involved in monogenic forms of diabetes and associated with *β*-cell development [125];* GRB14 *which is associated with obesity and insulin resistance [126]; and another three loci with less clear functions;* AP3S2*,* ST6GAL1* and* VPS26A* [92].

Another large meta-analysis in East Asian groups were performed in 2012 and identified 10 further novel loci [127] with mostly unknown function except for* GLIS3*, associated with *β*-cell development [128]. It is interesting that the* MAEA* variant discovered in this study is unique to East Asian and African populations, being monomorphic in Europeans and South Asians (Table 1; number 24) [129]. Several other variants identified in this study have substantially higher risk allele frequency (RAF) in East Asians than Europeans, for example,* ZFAND3* (Table 1; number 28; 34% versus 12%),* FITM2-R3HDML* (Table 1; number 111; 41% versus 18%), and* RPS3P7-MAF* (Table 2; number 56, 18% versus 1%), enhancing their detection in East Asian samples of relatively modest size.

Reflecting the success of initial T2D GWAS and the fast pace of technology, in 2012 Voight [130] and colleagues designed the “Metabochip,” a custom genotyping array enriched for variants shown to be associated with cardiometabolic traits via GWAS. These traits include T2D, coronary heart disease, myocardial infarction, body mass index, glucose and insulin level, lipid levels, and blood pressure. This new platform offers a powerful and cost-effective approach to both the discovery and follow-up of variants associated with these related traits, due to comprehensive coverage of previously associated loci (~120,000 SNPs) [130]. Morris and colleagues used the Metabochip to discover and characterize T2D-associated variants via meta-analysis of 34,840 cases and 114,981 controls of European descent, finding ten novel loci [131] not reported in previous European studies, all of which reached genome-wide significance. Another study using the Metabochip combined newly available samples with samples from previous discovery meta-analyses, using genotype data for 66,000 follow-up SNPs. This study identified 41 novel glycaemic associations, 33 of which were also associated with T2D [132]. This study implicated new loci in the aetiology of T2D and increased the overlap between loci associated with both glycaemia and T2D. These studies highlight the Metabochip as a promising tool for identifying novel and robustly associated loci, facilitating future research into underlying biology.

Taken together, the results of T2D GWAS signify tremendous progress in our quest to understand the genetic causes of T2D. Alternatively, they also highlight the genetic complexity of this disease. Genetic variants showing replicable association with T2D uniformly exert only a modest effect on disease risk, with per-allele odds ratios typically in the range of 1.1–1.3 (Table 1). The combined effect of all variants reported to date explains only about 10% of observed familial clustering [116]. Furthermore, the functional significance of various loci remains unclear. While some appear to be associated with *β*-cell function and insulin resistance, the biological role of many of them remains unknown. This suggests that the findings to date represent the first stage of a long journey to understanding T2D genetic risk.

### 2.7. Polygenic Models of T2D

The distribution of odds ratios observed for validated T2D-associated SNPs suggests that numerous, associated loci exist with even smaller effects than those identified to date. One would not expect such loci to have reached genome-wide significance in previous GWAS due to insufficient power. The existence of such additional small effect loci is consistent with the pattern of additional associated variants being discovered as sample sizes have increased; it is also consistent with validated SNP associations explaining only a small proportion of the T2D heritability estimated from twin studies, known as the “missing heritability” problem.

Two methods have recently been developed for assessing the contribution of common SNPs not reaching genome-wide significance to the heritability of a trait. These are polygenic scoring [133] and mixed linear modelling [134]. Both methods test the combined effects of thousands (or hundreds of thousands) of SNPs upon a trait of interest. A recent study by Stahl and colleagues used polygenic analyses and linear mixed modelling to show that thousands of SNPs contribute to T2D risk, estimating that about 50% of observed variance in T2D risk could be attributed to the combined effects of all SNPs genome-wide [135]. These investigators suggested that at least 70% of T2D heritability can be attributed to common SNPs represented on GWAS arrays [135], with most having very small individual effects upon disease risk.

### 2.8. Population Differences in T2D Risk Alleles

The frequency of T2D risk alleles often varies between populations, producing population differences in the attributable risk due to a particular genetic risk factor or combination of risk factors. The discovery of* KCNQ1* emphasized the impact of such frequency differences upon genetic discoveries [136]. Association of* KCNQ1* variants was found in East Asian populations [113, 114] using a considerably smaller sample size than that required to detect the association in with European populations [116], reflecting higher allele frequency (33% in East Asian versus 8% in Europeans) and hence statistical power in Asian groups. In addition, variants at the* TCF7L2* locus showed the inverse; a high risk allele frequency in Europeans (30%) compared to a low frequency in East Asians (3%) enhanced the detection in European studies [137]. Similarly, the* SYK* variant demonstrates a RAF of 26% in East Asians [138]and only 2% in Europeans and is monomorphic in Africans (Table 2; number 36). Further, a number of T2D risk variants are monomorphic (not variable) in some populations, preventing the detection of an association in these groups. The recently reported* SCGG* variant is unique to Indian Punjabi Sikh, being monomorphic in both European and African populations (Table 1; number 92). Other instances include the* THADA* variant, which was discovered in European populations but is monomorphic in East Asians (Table 1; number 4),* RND3-FABP5P10* that was discovered in African Americans but is monomorphic in Europeans (Table 1; number 6), and* G6PC2*, discovered in Europeans but monomorphic in Africans (Table 1; number 10). For a set of SNPs showing association with T2D across multiple populations, Table 3 shows risk allele frequencies and odds ratios for different populations in which associations have been reported. For these 14 SNPs, risk allele frequencies commonly differ across populations; however, allelic effects upon disease seem markedly consistent in both direction and magnitude, given overlapping confidence intervals for allelic odds ratios. Taken together, these results suggest that population differences can have important effects on power to* detect* common genetic associations for T2D in samples of diverse ancestry but may have less impact upon disease risk within individuals carrying the identified risk alleles. Nevertheless, at the population level, the attributable risk of such genetic variants will increase with allele frequency, thus potentially influencing population disease burden.

Significantly, a recent study assessing thousands of genetic associations showed that T2D has the most extreme population differentiation of risk allele frequencies among a broad range of complex diseases [139]. T2D risk allele frequencies demonstrated clear gradient matching paths of early human migration, suggesting potential differences in evolutionary adaptation to food availability, dietary patterns, or agricultural practices. This is consistent with “thrifty genotype” hypothesis [139, 140], which proposes that susceptibility to obesity and T2D in some populations reflects historical positive selection for genotypes promoting efficiency of metabolism, and energy and fat storage, thus providing an advantage in times of nutrient shortage [141]. This might explain the extraordinarily high prevalence of diabetes now seen among certain populations [34, 142, 143], potentially reflecting historical feast and famine cycles [62], increasing the frequency of thrifty genotypes and genetic predisposition to obesity and diabetes. While being unproven, this may partly explain higher susceptibility to abdominal obesity at lower BMI and reduced muscle mass with increased insulin resistance in Asian compared with Caucasian populations [7]. Nevertheless, pronounced population differentiation of T2D risk allele frequencies provides a strong rationale for further comprehensive genetic studies of T2D in diverse populations, expanding on the comprehensive studies in European samples.

To date, a range of non-European T2D GWAS have been conducted, including studies in Japanese [114, 119, 138, 144], Chinese [117, 145, 146], African-American [121, 147, 148], Southeast Asian [124], Hispanic [149], Mexican-American [150], South Asian [92], Indo-European [151], and Indian Punjabi Sikh [152]. These studies have led to new discoveries, including novel loci and loci that seem specific to certain populations [119, 127, 151, 152]. While many loci appear to contribute broadly to T2D risk, some loci have currently been confirmed in European populations only, including* WFS1*,* NOTCH2*,* THADA*,* ADAMTS9*,* TSPAN8/LGR5*,* INS-IGF2*,* ADCY5*,* GCK*,* MTRNR1B*,* HMGA2*,* HNF1A*,* ZBED3*,* KLF14*,* ZFAND6*,* PRC1*,* TLES/CHCHD9, *and* RBMS1 *[109, 116, 153–155]. Other loci currently show association specifically in East Asian populations, including* PTPRD*,* SRR*,* CDC123/CAMK1D*,* PSMD6*,* MAEA*,* ZFAND3*,* KCNK16*,* GCC1/PAX4*,* GLIS3*, and* PEPD *[117, 119, 120, 127]. On the other hand,* TMEM163 *[151] and* SGCG *[152] appear unique to South Asian and Indian Punjabi Sikh, respectively. Some of these discoveries may reflect the impact of population allele frequency differences, as previously discussed. In such cases, larger studies may eventually show that some loci contribute to disease across a broader range of populations.

Seemingly population-specific genetic associations for T2D may also reflect differences in the patterns of genomic correlation, or linkage disequilibrium (LD), between associated marker loci and the underlying unobserved functional variants. Populations with different demographic histories will often display different patterns of LD reflecting population differences in evolutionary recombination [156]. Older populations such as those in Africa have lower LD and can be helpful for finely localizing a risk variant following an initial association finding. This is because the genomic distance between disease-associated markers and true risk variants is likely to be smaller in such populations [157].

Thus, the apparent population-specificity of some known T2D risk alleles may reflect population differences in risk allele frequencies or LD between tagging and causal variants, rather than actual population-specificity of the underlying functional risk loci. We note that population-specific estimates of disease variance explained by all known T2D loci—although not widely reported—do seem largely similar between European and Asian populations. In their large 2012 study, Morris and colleagues [131] estimated that known common variants explain about 5.7% of T2D disease variance in Europeans. In 2013, Tabassum and colleagues [151] estimated that known loci combined with one novel Indian-specific locus explained 7.65% of T2D risk variance in South Asian Indians. The slightly higher estimate in Indians may potentially be explained by the inclusion of additional variants discovered between publications of the two studies, together with the inclusion of the Indian-specific locus discovered in the Tabassum study. Thus, available evidence thus does not strongly suggest that differences in the cumulative variance explained by known common T2D risk alleles can explain the markedly higher T2D prevalence observed in South Asians.

Interestingly, however, very recent studies show that population differences in linkage disequilibrium (LD) and the presence of multiple independent variants within a locus can markedly influence estimates of variance explained by known risk variants [158, 159]. Detailed fine mapping of T2D susceptibility loci in diverse populations, combined with the identification of underlying functional variants, may thus reveal population differences in the contribution of known loci to disease. Future research may also show the extent to which population differences in T2D risk can be explained by rare alleles, gene-environment interactions, or epigenetic effects.

### 2.9. Gene-Environment Interactions in T2D

In addition to the effects of specific genetic and environmental risk factors, gene-environment interactions are likely important mediators of population differences in T2D risk and contributors to the “missing heritability” problem. Indeed, given the relative stability of DNA sequence within populations over decades, recent increases in T2D prevalence must largely reflect environmental changes. Accordingly, the single largest contributor to T2D risk is obesity, and the global T2D epidemic chronologically parallels the global obesity epidemic.

A paucity of studies has examined gene-environment interactions in the context of T2D in general, let alone in Asian populations. A study by Qi and colleagues [160] found that a high genetic risk score formed from 10 T2D-associated SNPs was further increased by the presence of a “Westernized” dietary pattern characterised by increased red and processed meat intake and reduced dietary fibre [160]. The Westernized diet was not associated with increased risk among those with a low genetic risk score. Several studies have also found evidence for interactions between T2D-associated variants in* TCF7L2* and the quality and quantity of ingested carbohydrates in the context of T2D risk [161–163]. These studies support a possible contribution of gene-environment interactions to T2D risk, together with a potential model where interactions between recent lifestyle transitions and genetic risk factors may be contributing to the rapidly increasing prevalence of T2D in Asian populations. However, these preliminary findings require validation. Future analyses in well-designed, well-powered studies will help to clarify the role of gene-environment interactions in population differences in T2D risk.

### 2.10. Epigenetics

Similar to the “thrifty genotype” hypothesis, the “thrifty phenotype” hypothesis considers the adaptive consequences of the environment* in utero*. The hypothesis relates to the metabolic consequences of fetal malnutrition, proposing that adaptation to a low-calorie intrauterine environment induces permanent changes in chromatin structure and gene expression that influence insulin secretion and resistance, promoting more efficient energy utilisation and thus fetal survival [164]. According to the hypothesis, such epigenetic changes may predispose to insulin dysregulation, obesity, and T2D in later life. In support, epidemiologic studies have shown associations between small birth size, a marker for fetal malnutrition, and adult-onset T2D [165, 166]. A study by van Hoek and colleagues [167] in the Dutch Famine Birth Cohort detected an interaction between an* IGF2BP2* polymorphism and prenatal famine upon glucose level in the offspring. Interactions between other T2D risk variant alleles and birthweight have also been associated with increased T2D risk [168, 169].

## 3. Conclusions

We have discussed differences in prevalence, risk factor profiles, and genetic risk allele frequencies between different Asian countries and between Asian and other continental populations. Given these differences, continued T2D genetic studies in diverse populations are likely to contribute crucially to the broadening terrain of shared and unique population genetic effects for this disorder. Future studies will ideally include large, population-specific characterisation of risk variants, studies of gene-environment interaction, and epigenetic studies. Well-powered, well-designed studies performed in diverse Asian populations should enhance the benefits of genetic discoveries and their ultimate clinical translation for these large susceptible groups.

## Figures and Tables

**Table 1:** Published SNPs associated with Type 2 diabetes mellitus at genome-wide significance (*P* < 5 × 10^−8^).

Number	SNP (allele)^1,2^	Mapped gene(s)	Region^3^	Disc^4^ Pop	Rep^5^ Pop	RAF^6^	RAF_EU_^7^	RAF_EA_^8^	RAF_SA_^9^	RAF_AF_^10^	OR (95% CI)	*P* value
1	rs10923931 (T) [109]	*NOTCH2 *	1p12	EUR		0.11	0.09	0.03	0.22	0.39	1.13 (1.08–1.17)	4 × 10^−8^
2	rs340874 (C) [155]	*LINC00538-PROX1 *	1q32.3	EUR		0.52	0.56	0.32	0.55	0.08	NR	7 × 10^−12^
3	rs243021 (A) [116]	*FLJ30838-MIR4432 *	2p16.1	EUR	EUR	NR	0.48	0.64	0.48	0.45	1.08 (1.06–1.10)	3 × 10^−15^
4	rs7578597 (T) [109]	*THADA *	2p21	EUR		0.90	0.88	1.00	0.83	0.67	1.15 (1.10–1.20)	1 × 10^−9^
5	rs780094 (C) [155]	*GCKR *	2p23.3	EUR		0.62	0.61	0.43	0.80	0.88	NR	6 × 10^−38^
6	rs7560163 (C) [148]	*RND3-FABP5P10 *	2q23.3	AA		0.86	1.00	0.85	0.96	0.89	1.33 (1.19–1.49)	7 × 10^−9^
7	rs7593730 (C) [118]	*RBMS1 *	2q24.2	EUR		0.78	0.83	0.81	0.79	0.59	1.11 (1.08–1.16)	4 × 10^−8^
8	rs3923113 (A) [92]	*EIF3EP3-SNORA70F *	2q24.3	SA		0.74	0.59	0.84	0.76	0.23	1.09 (1.06–1.13)	1 × 10^−8^
9	rs13389219 (C) [131]	*GRB14-COBLL1 *	2q24.3	EUR		0.60	0.56	0.79	—	0.15	1.07 (1.05–1.10)	1.0 × 10^−8^
10	rs560887 (C) [155]	*G6PC2 *	2q31.1	EUR		0.70	0.67	0.98	0.85	1.00	NR	9 × 10^−218^
11	rs7578326 (A) [116]	*LOC646736 *	2q36.3	EUR		NR	0.65	0.87	0.85	0.60	1.11 (1.08–1.13)	5 × 10^−20^
12	rs2943641 (C) [115]	*NYAP2-MIR5702 *	2q36.3	EUR	EUR	0.63	0.63	0.94	0.74	0.76	1.19 (1.13–1.25)	9 × 10^−12^
13	rs4607103 (C) [109]	*ADAMTS9-AS2 *	3p14.1	EUR		0.76	0.81	0.56	0.48	0.71	1.09 (1.06–1.12)	1 × 10^−8^
14	rs831571 (C) [127]	*PSMD6-PRICKLE2-AS1 *	3p14.1	EA		0.61	0.77	0.65	0.83	0.84	1.09 (1.06–1.12)	8 × 10^−11^
15	rs11708067 (A) [155]	*ADCY5 *	3q21.1	EUR		0.78	0.77	0.99	0.77	0.89	NR	7 × 10^−22^
16	rs11920090 (T) [155]	*SLC2A2 *	3q26.2	EUR		0.87	0.86	0.98	—	0.68	NR	8 × 10^−13^
17	rs1470579 (C) [116]	*IGF2BP2 *	3q27.2	EUR	SA, EA, PS	NR	0.30	0.24	0.50	0.86	1.14 (1.09–1.19)	2 × 10^−9^
18	rs6769511 (C) [114]	*IGF2BP2 *	3q27.2	EA		0.32	0.30	0.24	0.50	0.84	1.23 (1.15–1.31)	1 × 10^−9^
19	rs4402960 (T) [98]	*IGF2BP2 *	3q27.2	EUR	EA	0.30	0.30	0.22	0.49	0.54	1.14 (1.11–1.18)	9 × 10^−16^
20	rs16861329 (G) [92]	*ST6GAL1 *	3q27.3	SA		0.75	0.88	0.80	0.76	0.94	1.09 (1.06–1.12)	3 × 10^−8^
21	rs1801214 (T) [116]	*WFS1 *	4p16.1	EUR		NR	0.12	0.20	0.24	0.06	1.13 (1.08–1.18)	3 × 10^−8^
22	rs4689388 (T) [115]	*WFS1 *	4p16.1	EUR	EUR	0.57	0.67	0.97	0.68	0.72	1.16 (1.10–1.21)	1 × 10^−8^
23	rs7656416 (C) [138]	*CTBP1-AS1-MAEA *	4p16.3	EA		0.68	—	0.70	—	0.90	1.15 (1.10–1.21)	1 × 10^−8^
24	rs6815464 (C) [127]	*MAEA *	4p16.3	EA		0.58	—	0.52	—	0.92	1.13 (1.10–1.16)	2 × 10^−20^
25	rs459193 (G) [131]	*ANKRD55-MAP3K1 *	5q11.2	EUR		0.70	0.78	0.51	—	0.64	1.08 (1.05–1.11)	6.0 × 10^−9^
26	rs4457053 (G) [116]	*ZBED3-AS1 *	5q13.3	EUR		NR	0.26	0.04	—	—	1.08 (1.06–1.11)	3 × 10^−12^
27	rs1535500 (T) [127]	*KCNK16; KCNK17 *	6p21.2	EA		0.42	0.47	0.44	—	0.95	1.08 (1.05–1.11)	2 × 10^−8^
28	rs9470794 (C) [127]	*ZFAND3 *	6p21.2	EA		0.27	0.12	0.34	0.14	0.19	1.12 (1.08–1.16)	2 × 10^−10^
29	rs10440833 (A) [116]	*CDKAL1 *	6p22.3	EUR	EUR	NR	0.25	0.39	—	0.21	1.25 (1.20–1.31)	2 × 10^−22^
30	rs4712523 (G) [144]	*CDKAL1 *	6p22.3	EA	EUR	0.41	0.34	0.41	0.24	0.68	1.27 (1.21–1.33)	7 × 10^−20^
31	rs10946398 (C)[74]	*CDKAL1 *	6p22.3	EUR		0.32	0.34	0.40	0.22	0.67	1.16 (1.10–1.22)	1 × 10^−8^
32	rs6931514 (G) [109]	*CDKAL1 *	6p22.3	EUR		NR	0.28	—	0.22	0.22	1.25 (1.17–1.33)	1 × 10^−11^
33	rs7754840 (C) [103]	*CDKAL1 *	6p22.3	EUR	EA	0.31	0.34	0.40	0.22	0.67	1.12 (1.08–1.16)	4 × 10^−11^
34	rs7756992 (G) [170]	*CDKAL1 *	6p22.3	EUR		0.26	0.28	0.50	0.24	0.58	1.20 (1.13–1.27)	8 × 10^−9^
35	rs7766070 (A) [171]	*CDKAL1 *	6p22.3	EUR		0.27	0.25	0.40	—	0.19	1.21 (1.14–1.28)	6 × 10^−11^
36	rs1048886 (G) [124]	*C6orf57 *	6q13	SEA (I)		0.18	0.15	0.09	0.17	0.34	1.54 (1.32–1.80)	3 × 10^−8^
37	rs4607517 (A) [155]	*GCK-YKT6 *	7p13	EUR		0.16	0.20	0.22	0.12	0.06	NR	7 × 10^−92^
38	rs849134 (A) [116]	*JAZF1 *	7p15.1	EUR		NR	0.54	0.81	—	0.77	1.13 (1.09–1.18)	3 × 10^−9^
39	rs864745 (T) [109]	*JAZF1 *	7p15.1	EUR		0.50	0.49	0.77	0.79	0.77	1.10 (1.07–1.13)	5 × 10^−14^
40	rs2191349 (T) [155]	*DGKB-AGMO *	7p21.2	EUR		0.52	0.48	0.71	—	0.58	NR	3 × 10^−44^
41	rs10229583 (G) [172]	*FSCN3-PAX4 *	7q32	EA		0.83	0.74	0.82	0.66	0.72	1.14 (1.09–1.19)	2 × 10^−10^
42	rs6467136 (G) [127]	*ZNF800-GCC1 *	7q32.1	EA		0.79	0.50	0.76	0.57	0.75	1.11 (1.07–1.14)	5 × 10^−11^
43	rs972283 (G) [116]	*KLF14-MIR29A *	7q32.3	EUR		NR	0.55	0.67	—	0.94	1.07 (1.05–1.10)	2 × 10^−10^
44	rs516946 (C) [131]	*ANK1 *	8p11.21	EUR		0.76	0.81	0.82	0.85	0.88	1.09 (1.06–1.12)	2.5 × 10^−10^
45	rs515071 (G) [138]	*ANK1; MIR486 *	8p11.21	EA		0.79	0.81	0.79	0.85	0.85	1.18 (1.12–1.25)	1 × 10^−8^
46	rs896854 (T) [116]	*TP53INP1 *	8q22.1	EUR		NR	0.44	0.32	0.40	0.75	1.06 (1.04–1.09)	1 × 10^−9^
47	rs3802177 (G) [116]	*SLC30A8 *	8q24.11	EUR		NR	0.76	0.53	0.78	0.94	1.15 (1.10–1.21)	1 × 10^−8^
48	rs11558471 (A) [155]	*SLC30A8 *	8q24.11	EUR		0.31	0.75	0.53	0.78	0.94	NR	3 × 10^−11^
49	rs13266634 (C) [144]	*SLC30A8 *	8q24.11	EUR	EA	0.57	0.76	0.53	0.78	0.94	1.22 (1.16–1.28)	2 × 10^−14^
50	rs10965250 (G) [116]	*CDKN2B-AS1-DMRTA1 *	9p21.3	EUR	EUR	NR	0.80	0.58	—	1.00	1.20 (1.13–1.27)	1 × 10^−10^
51	rs1333051 (A) [149]	*CDKN2B-AS1-DMRTA1 *	9p21.3	HIS		NR	0.84	0.85	0.93	0.91	1.22 (1.15–1.30)	6 × 10^−10^
52	rs2383208 (A) [144]	*CDKN2B-AS1-DMRTA1 *	9p21.3	EA	EA, SA	0.55	0.79	0.59	0.91	0.87	1.34 (1.27–1.41)	2 × 10^−29^
53	rs10811661 (T) [98]	*CDKN2B-AS1-DMRTA1 *	9p21.3	EUR		0.85	0.80	0.56	0.91	0.98	1.20 (1.14–1.25)	8 × 10^−15^
54	rs7018475 (G) [173]*	*CDKN2B-AS1-DMRTA1 *	9p21.3	EUR		NR	0.27	0.37	0.38	0.22	1.35 (1.18–1.56)	3 × 10^−8^
55	rs17584499 (T) [117]	*PTPRD *	9p24.1	EA		0.06	0.23	0.11	0.25	0.03	1.57 (1.36–1.82)	9 × 10^−10^
56	rs10814916 (C) [146]	*GLIS3 *	9p24.2	EA		0.44	0.57	0.45	0.62	0.67	1.11 (1.08–1.15)	6 × 10^−12^
57	rs7041847 (A) [127]	*GLIS3 *	9p24.2	EA		0.41	0.55	0.46	0.65	0.96	1.10 (1.07–1.13)	2 × 10^−14^
58	rs7034200 (A) [155]	*GLIS3 *	9p24.2	EUR		0.49	0.54	0.30	—	0.60	NR	1 × 10^−12^
59	rs13292136 (C) [116]	*KRT18P24-CHCHD2P9 *	9q21.31	EUR	EUR	NR	0.93	0.91	—	0.90	1.11 (1.07–1.15)	3 × 10^−8^
60	rs2796441 (G) [131]	*TLE1-FAM75D5 *	9q21.32	EUR		0.57	0.61	0.40	0.55	0.90	1.07 (1.05–1.10)	5.4 × 10^−9^
61	rs10906115 (A) [120]	*CDC123-MIR4480 *	10p13	EA		0.57	0.64	0.64	0.58	0.76	1.13 (1.08–1.18)	1 × 10^−8^
62	rs11257655 (T) [146]	*CDC123-MIR4480 *	10p13	EA		0.56	0.26	0.58	0.23	0.27	1.15 (1.10–1.20)	7 × 10^−9^
63	rs12779790 (G) [109]	*CDC123-MIR4480 *	10p13	EUR		0.18	0.22	0.13	—	0.05	1.11 (1.07–1.14)	1 × 10^−10^
64	rs1802295 (A) [92]	*VPS26A *	10q22.1	SA		0.26	0.35	0.11	0.29	0	1.08 (1.05–1.12)	4 × 10^−8^
65	rs12571751 (A) [131]	*ZMIZ1 *	10q22.3	EUR		0.52	0.53	0.55	0.50	0.50	1.08 (1.05–1.10)	1 × 10^−10^
66	rs1111875 (C) [98]	*HHEX-EXOC6 *	10q23.33	EUR	EA	0.52	0.58	0.34	0.41	0.88	1.13 (1.09–1.17)	6 × 10^−10^
67	rs5015480 (C) [171]	*HHEX-EXOC6 *	10q23.33	EUR	EA	0.57	0.58	0.21	0.44	0.64	1.18 (1.11–1.23)	2 × 10^−9^
68	rs10885122 (G) [155]	*ADRA2A-BTBD7P2 *	10q25.2	EUR		0.87	0.90	0.92	—	0.21	NR	3 × 10^−16^
69	rs4506565 (T) [104]	*TCF7L2 *	10q25.2	EUR		0.32	0.30	0.03	0.30	0.46	1.36 (1.20–1.54)	5 × 10^−12^
70	rs7901695 (C) [74]	*TCF7L2 *	10q25.2	EUR		NR	0.28	0.03	0.29	0.46	1.37 (1.31–1.43)	1 × 10^−48^
71	rs7903146 (T) [116]	*TCF7L2 *	10q25.2	EUR	EA, SA, AA	NR	0.28	0.03	0.28	0.27	1.40 (1.34–1.46)	2 × 10^−51^
72	rs10886471 (C) [146]	*GRK5 *	10q26.11	EA		0.78	0.48	0.80	0.65	0.90	1.12 (1.08–1.16)	7 × 10^−9^
73	rs11605924 (A) [155]	*CRY2 *	11p11.2	EUR		0.49	0.13	0.70	0.12	0.94	NR	1 × 10^−14^
74	rs7944584 (A) [155]	*MADD *	11p11.2	EUR		0.75	0.71	0.96	0.78	1.00	NR	2 × 10^−18^
75	rs5215 (C) [74]	*KCNJ11 *	11p15.1	EUR		NR	0.40	0.38	0.4	0.01	1.14 (1.10–1.19)	5 × 10^−11^
76	rs5219 (T) [98]	*KCNJ11 *	11p15.1	EUR		0.46	—	—	—	—	1.14 (1.10–1.19)	7 × 10^−11^
77	rs163182 (C) [145]	*KCNQ1 *	11p15.4	EA		0.34	0.25	0.37	—	0.25	1.28 (NR)	2 × 10^−17^
78	rs2237895 (C) [117]	*KCNQ1 *	11p15.4	EA		0.33	—	—	—	—	1.29 (1.19–1.40)	1 × 10^−9^
79	rs2237892 (C) [113]	*KCNQ1 *	11p15.4	EA	HIS, EA	0.61	0.92	0.67	0.99	0.90	1.40 (1.34–1.47)	2 × 10^−42^
80	rs2237897 (C) [114]	*KCNQ1 *	11p15.4	EA		0.34	0.95	—	—	—	1.33 (1.24–1.41)	1 × 10^−16^
81	rs231362 (G) [116]	*KCNQ1; KCNQ1OT1 *	11p15.5	EUR		NR	0.52	0.84	—	0.86	1.08 (1.06–1.10)	3 × 10^−13^
82	rs174550 (T) [155]	*FADS1 *	11q12.2	EUR		0.64	0.65	0.66	—	0.98	NR	2 × 10^−15^
83	rs1552224 (A) [116]	*ARAP1 *	11q13.4	EUR		NR	0.87	0.91	0.76	1.00	1.14 (1.11–1.17)	1 × 10^−22^
84	rs1387153 (T) [116]	*FAT3-MTNR1B *	11q14.3	EUR	EUR	NR	0.27	0.39	0.38	0.40	1.09 (1.06–1.11)	8 × 10^−15^
85	rs10830963 (G) [155]	*MTNR1B *	11q14.3	EUR		0.30	0.30	0.39	—	0.04	NR	6 × 10^−175^
86	rs10842994 (C) [131]	*KLHDC5 *	12p11.22	EUR		0.80	0.80	0.79	0.90	1.00	1.10 (1.06–1.13)	6.1 × 10^−10^
87	rs1531343 (C) [116]	*RPSAP52 *	12q14.3	EUR		NR	0.12	0.08	0.19	0.40	1.10 (1.07–1.14)	4 × 10^−9^
88	rs7961581 (C) [109]	*TSPAN8-LGR5 *	12q21.1	EUR		0.27	0.25	0.17	0.35	0.18	1.09 (1.06–1.12)	1 × 10^−9^
89	rs35767 (G) [155]	*IGF1 *	12q23.2	EUR		0.85	0.88	0.65	0.71	0.55	NR	3 × 10^−8^
90	rs7957197 (T) [116]	*OASL *	12q24.31	EUR		NR	0.85	1.00	—	0.86	1.07 (1.05–1.10)	2 × 10^−8^
91	rs7305618 (C) [149]	*RPL12P33-HNF1A-AS1 *	12q24.31	HIS		NR	0.80	0.44	0.75	0.56	1.14 (1.09–1.20)	2 × 10^−8^
92	rs9552911 (G) [152]	*SGCG *	13q12.12	PS	PS	0.93	1.00	0.78	0.86	1.00	1.49 (1.30–1.72)	2 × 10^−8^
93	rs1359790 (G) [120]	*NDFIP2-SPRY2 *	13q31.1	EA		0.71	0.73	0.69	0.84	0.92	1.15 (1.10–1.20)	6 × 10^−9^
94	rs7403531 (T) [146]	*RASGRP1 *	15q14	EA		0.35	0.28	0.33	0.20	0.18	1.10 (1.06–1.13)	4 × 10^−9^
95	rs7172432 (A) [119]	*C2CD4A-C2CD4B *	15q22.2	EA		0.58	0.58	0.54	0.61	0.27	1.11 (1.08–1.14)	9 × 10^−14^
96	rs11071657 (A) [155]	*C2CD4A-C2CD4B *	15q22.2	EUR		0.63	0.58	0.69	—	0.94	NR	4 × 10^−8^
97	rs7178572 (G) [92]	*HMG20A *	15q24.3	SA	EUR	0.52	0.68	0.40	0.44	0.40	1.09 (1.06–1.12)	7 × 10^−11^
98	rs7177055 (A) [131]	*HMG20A-LINGO1 *	15q24.3	EUR		0.68	0.71	0.39	0.45	0.24	1.08 (1.05–1.10)	4.6 × 10^−9^
99	rs11634397 (G) [116]	*ZFAND6-FAH *	15q25.1	EUR	EUR	NR	0.64	0.08	0.55	0.41	1.06 (1.04–1.08)	2 × 10^−9^
100	rs2028299 (C) [92]	*AP3S2; C15orf38-AP3S2 *	15q26.1	SA		0.31	0.73	0.12	0.78	0.40	1.10 (1.07–1.13)	2 × 10^−11^
101	rs8042680 (A) [116]	*PRC1; LOC100507118 *	15q26.1	EUR		NR	0.26	1.00	0.59	0.98	1.07 (1.05–1.09)	2 × 10^−10^
102	rs11642841 (A) [116]	*FTO *	16q12.2	EUR	EUR	NR	0.47	0.06	—	0.04	1.13 (1.08–1.18)	3 × 10^−8^
103	rs8050136 (A) [98]	*FTO *	16q12.2	EUR	SA	0.38	0.46	0.14	0.25	0.45	1.17 (1.12–1.22)	1 × 10^−12^
104	rs9939609 (A) [171]	*FTO *	16q12.2	EUR		0.40	0.46	0.15	0.26	0.50	1.25 (1.19–1.30)	1 × 10^−20^
105	rs7202877 (T) [131]	*CTRB2-CTRB1 *	16q23.1	EUR		0.89	0.89	0.80	0.95	0.85	1.12 (1.07–1.16)	3.5 × 10^−8^
106	rs391300 (G) [117]	*SRR *	17p13.3	EA		0.62	0.63	0.75	0.48	0.42	1.28 (1.18–1.39)	3 × 10^−9^
107	rs4430796 (G) [146]	*HNF1B *	17q12	EUR	EA	0.28	0.51	0.25	0.31	0.66	1.19 (1.13–1.25)	2 × 10^−11^
108	rs8090011 (G) [171]	*LAMA1 *	18p11.31	EUR		0.38	0.32	0.72	—	0.79	1.13 (1.09–1.18)	8 × 10^−9^
109	rs12970134 (A) [131]	*MC4R *	18q21.32	EUR		0.27	0.28	0.18	0.29	0.17	1.08 (1.05–1.11)	1.2 × 10^−8^
110	rs3786897 (A) [127]	*PEPD *	19q13.11	EA		0.56	0.61	0.58	0.81	0.40	1.10 (1.07–1.14)	1 × 10^−8^
111	rs6017317 (G) [127]	*FITM2-R3HDML *	20q13.12	EA		0.48	0.18	0.41	—	0.59	1.09 (1.07–1.12)	1 × 10^−11^
112	rs4812829 (A) [92]	*HNF4A *	20q13.12	SA		0.29	0.16	0.45	0.29	0.08	1.09 (1.06–1.12)	3 × 10^−10^
113	rs12010175 (G) [146]	*FAM58A *	Xq28	EA		0.79	0.94	0.84	0.81	0.79	1.21 (1.14–1.28)	2 × 10^−9^
114	rs5945326 (A) [116]	*KRT18P48-DUSP9 *	Xq28	EUR	EA	NR	0.78	0.66	—	0.84	1.27 (1.18–1.37)	3 × 10^−10^

*Risk Allele and RAF not reported, but chosen based on minor allele frequency (MAF) in the population mentioned in the original publication.

**Table 2:** Published SNPs associated with Type 2 diabetes mellitus at suggestive significance (*P* < 1 × 10^−5^).

Number	SNP (allele)^1,2^	Mapped Gene(s)	Region^3^	Disc^4^ Pop	Rep^5^ Pop	RAF^6^	RAF_EU_^7^	RAF_EA_^8^	RAF_SA_^9^	RAF_AF_^10^	OR (95% CI)	*P* value
1	rs7542900 (C) [148]	*F3-PGBD4P7 *	1p21.3	AA		0.56	0.80	0.72	0.79	0.56	1.16 (1.09–1.25)	6 × 10^−6^
2	rs11165354 (A) [151]	*TGFBR3 *	1p22.1	SA	All SA	0.78	0.62	0.52	0.85	0.28	1.17 (1.10–1.25)	4 × 10^−6^
3	rs17045328 (G) [124]	*CR2 *	1q32.2	SEA (M)		0.30	0.03	0.35	0.07	0.02	1.38 (1.20–1.59)	7 × 10^−6^
4	rs6426514 (A) [152]	*RHOU *	1q42.13	PS		0.06	0.09	0.03	0.02	0.12	1.51 (1.27–1.78)	2 × 10^−6^
5	rs12027542 (A) [124]	*PCNXL2 *	1q42.2	SEA (M)		0.61	0.93	0.69	0.95	0.95	1.41 (1.23–1.61)	4 × 10^−7^
6	rs11677370 (T)[124]	*DCDC2C *	2p25.3	SEA (I)		0.4	0.68	0.71	—	0.78	1.35 (1.19–1.53)	3 × 10^−6^
7	rs6712932 (C) [174]*	*MRPS9-GPR45 *	2q12.1	EUR		NR	0.34	0.22	0.32	0.28	1.52 (1.27–1.82)	6 × 10^−6^
8	rs6723108 (T) [151]	*TMEM163-MIR5590 *	2q21.3	SA	All SA	0.86	0.50	1	0.93	1	1.27 (1.17–1.39)	7 × 10^−8^
9	rs358806 (C) [104]	*LRTM1-WNT5A *	3p14.3	EUR		0.80	0.77	0.84	0.90	0.92	1.16 (1.03–1.33)	3 × 10^−6^
10	rs13081389 (A) [116]	*SYN2-GSTM5P1 *	3p25.2	EUR		NR	0.95	0.98	—	1.00	1.24 (1.15–1.35)	2 × 10^−7^
11	rs17036101 (G) [109]	*SYN2-GSTM5P1 *	3p25.2	EUR		0.93	0.95	0.98	—	0.98	1.15 (1.10–1.21)	2 × 10^−7^
12	rs1801282 (C) [103]	*PPARG *	3p25.2	EUR		0.86	0.90	0.94	0.91	1.00	1.14 (1.08–1.20)	2 × 10^−6^
13	rs2063640 (A) [124]	*ZPLD1-NDUFA4P2 *	3q12.3	SEA (M, C, I)		0.17	0.08	0.27	0.11	0.04	1.23 (1.13–1.34)	3 × 10^−6^
14	rs3773506 (C) [124]	*PLS1 *	3q23	SEA (I)		0.06	0.04	0.11	0.04	0.14	1.81 (1.39–2.35)	9 × 10^−6^
15	rs7630877 (A) [124]	*PEX5L *	3q26.33	SEA (C)		0.17	0.35	0.18	0.36	0.31	1.32 (1.17–1.49)	7 × 10^−6^
16	rs1374910 (T) [149]	*IGF2BP2 *	3q27.2	HIS		NR	0.02	0.08	—	0.15	1.24 (1.15–1.34)	1 × 10^−7^
17	rs7659604 (T) [104]	*ANXA5-TMEM155 *	4q27	EUR		0.38	0.44	0.36	0.45	0.71	1.35 (1.19–1.54)	9 × 10^−6^
18	rs3792615 (T) [124]	*36951 *	4q32.3	SEA (I)		0.95	0.97	0.84	0.96	0.85	1.93 (1.45–2.59)	9 × 10^−6^
19	rs10461617 (A) [151]	*RPL26P19-MAP3K1 *	5q11.2	SA	All SA	0.21	0.18	0.39	0.26	0.44	1.17 (1.09–1.25)	4 × 10^−6^
20	rs12518099 (C) [115]	*MIR3660-CETN3 *	5q14.3	EUR		0.23	0.23	0.39	0.31	0.23	1.16 (1.10–1.22)	7 × 10^−7^
21	rs17053082 (T) [152]	*PPIGP1-SGCD *	5q33.2	PS		0.1	0.06	0.06	0.06	0.08	1.49 (1.28–1.73)	4 × 10^−7^
22	rs9472138 (T) [109]	*VEGFA-C6orf223 *	6p21.1	EUR		0.28	0.24	0.11	0.23	0.14	1.06 (1.04–1.09)	4 × 10^−6^
23	rs3916765 (A) [171]	*MTCO3P1-HLA-DQA2 *	6p21.32	EUR		0.12	0.17	0.08	0.08	0	1.21 (1.12–1.31)	1 × 10^−6^
24	rs9295474 (G) [124]	*CDKAL1 *	6p22.3	SEA (M, C, I)		0.36	0.30	0.41	—	0.36	1.16 (1.09–1.24)	9 × 10^−6^
25	rs9465871 (C) [104]	*CDKAL1 *	6p22.3	EUR		0.18	0.16	0.52	0.21	0.58	1.18 (1.04–1.34)	3 × 10^−7^
26	rs7769051 (A) [121]	*SNORA33-HMGB1P13 *	6q23.2	AA		0.29	0.10	0.04	0.16	0.38	1.28 (1.16–1.42)	2 × 10^−6^
27	rs642858 (A) [124]	*ATP5F1P6-MIR3668 *	6q24.1	SEA (I)		0.40	0.25	0.40	0.36	0.13	1.35 (1.19–1.53)	2 × 10^−6^
28	rs6930576 (A) [121]	*SASH1 *	6q24.3	AA		0.28	0.34	0.18	0.44	0.24	1.31 (1.18–1.45)	7 × 10^−7^
29	rs741301 (C) [175]*	*ELMO1 *	7p14.2	EA		NR	0.32	0.32	0.43	0.67	2.67 (1.71–4.16)	8 × 10^−6^
30	rs1525739 (C) [176]*	*LOC100287613 *	7p21	EUR		NR	0.49	0.16	0.27	0.33	NR	6 × 10^−6^
31	rs7636 (A) [124]	*ACHE *	7q22.1	EA		0.06	0.04	0	0.05	0.33	1.85 (1.42–2.41)	5 × 10^−6^
32	rs4527850 (T) [152]	*SLA-WISP1 *	8q24.22	PS		0.75	0.72	0.42	0.69	0.89	1.23 (1.13–1.34)	2 × 10^−6^
33	rs564398 (T) [74]	*CDKN2B-AS1 *	9p21.3	EUR		0.56	0.57	0.92	0.67	1.00	1.13 (1.08–1.19)	1 × 10^−6^
34	rs7020996 (C) [109]	*CDKN2B-AS1-DMRTA1 *	9p21.3	EUR		NR	0.81	0.57	—	0.74	1.26 (1.15–1.38)	2 × 10^−7^
35	rs649891 (C) [150]	*PTPRD *	9p23	MA		0.35	0.20	0.73	0.43	0.79	NR	6 × 10^−6^
36	rs10993738 (C) [138]	*SYK *	9q22.2	EA		0.15	0.02	0.26	—	0	1.16 (1.09–1.23)	5 × 10^−6^
37	rs773506 (G) [121]	*SYK-AUH *	9q22.31	AA		0.77	0.63	0.25	0.50	0.13	1.32 (1.18–1.49)	6 × 10^−6^
38	rs10980508 (A) [176]*	*SVEP1-RPS21P5 *	9q31	EUR		NR	0.86	0.97	0.97	0.94	NR	1 × 10^−6^
39	rs1327796 (G) [138]	*PALM2 *	9q31.3	EA		0.24	0.22	0.27	—	0.21	1.13 (1.08–1.20)	3 × 10^−6^
40	rs6583826 (G) [124]	*IDE-RPL11P4 *	10q23.33	SEA (M, C, I)		0.26	0.53	0.27	0.33	0.50	1.18 (1.10–1.27)	7 × 10^−6^
41	rs10741243 (G) [124]	*TCERG1L *	10q26.3	SEA (I)		0.93	0.95	0.89	—	0.49	1.75 (1.38–2.23)	5 × 10^−6^
42	rs9300039 (C) [98]	*RPL9P23-HNRNPKP3 *	11p12	EUR		0.89	0.87	0.70	0.82	0.85	1.48 (1.28–1.71)	6 × 10^−8^
43	rs2722769 (C) [148]	*HMGN1P22-MTND5P21 *	11p15.3	AA		0.53	0.56	0.56	0.76	0.99	1.35 (1.19–1.54)	2 × 10^−6^
44	rs7107217 (C) [148]	*RPS27P20-TMEM45B *	11q24.3	AA		0.91	0.50	0.34	0.65	0.54	1.18 (1.10–1.27)	3 × 10^−7^
45	rs12304921 (G) [104]	*HIGD1C *	12q13.12	EUR		0.15	0.16	0.50	0.36	0.15	2.5 (1.53–4.09)	7 × 10^−6^
46	rs1153188 (A) [109]	*DCD-VDAC1P5 *	12q13.2	EUR		0.73	0.74	0.99	0.83	0.79	1.08 (1.05–1.11)	2 × 10^−7^
47	rs2358944 (G) [121]	*PCNPP3-RPSAP52 *	12q14.3	AA		0.77	0.14	0.67	0.38	0.89	1.33 (1.18–1.49)	4 × 10^−6^
48	rs1495377 (G) [104]	*TSPAN8-LGR5 *	12q21.1	EUR		0.50	0.48	0.24	0.41	0.15	1.28 (1.11–1.49)	7 × 10^−6^
49	rs4760790 (A) [116]	*TSPAN8-LGR5 *	12q21.1	EUR		NR	0.22	0.24	—	0.14	1.11 (1.06–1.16)	4 × 10^−6^
50	rs730570 (G) [149]	*BEGAIN-DLK1 *	14q32.2	HIS		NR	0.16	0.80	0.45	0.80	1.14 (1.08–1.21)	8 × 10^−6^
51	rs1436953 (G) [145]	*C2CD4A-C2CD4B *	15q22.2	EA		0.64	0.43	0.57	0.57	0.24	1.14 (NR)	8 × 10^−6^
52	rs1436955 (C) [120]	*C2CD4A-C2CD4B *	15q22.2	EA		0.73	0.74	0.74	0.75	0.65	1.13 (1.08–1.19)	7 × 10^−7^
53	rs7119 (T) [124]	*HMG20A *	15q24.3	SEA (M, C, I)		0.19	0.40	0.17	0.24	0.38	1.24 (1.14–1.34)	5 × 10^−7^
54	rs17177078 (C) [176]*	*TNRC6A *	16p12	EUR		NR	0.93	1.00	0.97	1.00	NR	5 × 10^−6^
55	rs16955379 (C) [127]	*CMIP *	16q23.2	EA		0.80	0.98	0.77	—	0.96	1.08 (1.05–1.12)	3 × 10^−7^
56	rs17797882 (T) [127]	*RPS3P7-MAF *	16q23.2	EA		0.32	0.01	0.18	0.04	0.05	1.08 (1.05–1.12)	9 × 10^−7^
57	rs623323 (T) [152]	*RNMTL1-NXN *	17p13.3	PS		0.15	0.20	0.11	0.13	0.50	1.28 (1.15–1.42)	4 × 10^−6^
58	rs10460009 (C) [124]	*LPIN2; LOC727896 *	18p11.31	SEA (M)		0.60	0.92	0.53	0.73	0.95	1.35 (1.18–1.54)	9 × 10^−6^
59	rs472265 (G) [124]	*PAPL *	19q13.2	SEA (I)		0.22	0.16	0.26	0.27	0.26	1.39 (1.20–1.61)	9 × 10^−6^
60	rs328506 (C) [152]	*RBM38-HMGB1P1 *	20q13.31	PS	SA	0.80	0.72	1.00	0.90	0.72	1.11 (1.06–1.15)	2 × 10^−6^
61	rs2833610 (A) [124]	*HUNK-MIS18A *	21q22.11	SEA (M, C, I)		0.57	0.30	0.57	0.51	0.32	1.17 (1.09–1.24)	4 × 10^−6^
62	rs2106294 (T) [121]	*LIMK2 *	22q12.2	AA		0.94	0.70	0.86	0.75	1.00	1.75 (1.39–2.22)	4 × 10^−6^
63	rs470089 (G) [176]*	*SULT4A1 *	22q13.3	EUR		NR	0.8	0.93	0.76	0.60	NR	9 × 10^−6^

^1^ *Th* *e* SNP-risk allele: SNP(s) most strongly associated with trait (risk allele).

^2^Reference for the largest study reporting association of the SNP with T2D or fasting plasma glucose at genome-wide significance (*P* < 5 × 10^−8^).

^3^Cytogenetic region associated with the SNP (NCBI).

^4^Discovery population; EUR: European; SA: South Asian; EA: East Asian; SEA: Southeast Asian; AA: African-American, MA: Mexican-American; HIS: Hispanic; PS: Punjabi Sikh; M: Malay; C: Chinese; I: Indian.

^5^Replication population: it has been confirmed in other populations; EUR: European; SA: South Asian; EA: East Asian; SEA: Southeast Asian; AA: African- American, MA: Mexican American; HIS: Hispanic; PS: Punjabi Sikh; M: Malay; C: Chinese; I: Indian.

^6^Reported risk allele frequency (RAF) for the SNP; NR if not reported.

^7^RAF in HapMap population for Utah residents with Northern and Western European-ancestry from the CEPH collection; “—” denotes data not listed in HapMap.

^8^RAF in HapMap population for Han Chinese in Beijing, China; “—” denotes data not listed in HapMap.

^9^RAF in HapMap population for Gujarati Indians in Houston, Texas; “—” denotes data not listed in HapMap.

^10^RAF in HapMap population for Yoruban in Ibadan, Nigeria; “—” denotes data not listed in HapMap.

*Risk Allele and RAF not reported, but chosen based on minor allele frequency (MAF) in the population mentioned in the original publication.

**Table 3:** Population-specific odds ratios and risk allele frequencies for SNPs associated with T2D in multiple populations.

Number	SNPs	Ref allele	Disc Pop	RAF	OR (95% CI)	Ref allele	1st Rep Pop	RAF	OR (95% CI)	Ref allele	2nd Rep Pop	RAF	OR (95% CI)
1	rs1470579	C	EUR [116]	0.3	1.14 (1.09–1.19)	C	EA [177]	0.24	1.33 (1.20–1.48)	C	SA [152]	0.5	1.06 (1.04–1.08)
2	rs4402960	T	EUR [98]	0.3	1.14 (1.11–1.18)	T	EA [144]	0.22	1.14 (1.08–1.21)				
3	rs7754840	C	EUR [103]	0.34	1.12 (1.08–1.16)	C	EA [146]	0.4	1.35 (1.23–1.48)				
4	rs13266634	C	EUR [74]	0.76	1.12 (1.07–1.16)	C	EA [144]	0.53	1.22 (1.15–1.28)				
5	rs2383208	A	EA [144]	0.59	1.34 (1.27–1.41)	A	SA [151]	0.91	1.23 (1.13–1.34)				
6	rs1111875	C	EUR [98]	0.58	1.13 (1.09–1.17)	C	EA [144]	0.34	1.21 (1.15–1.28)				
7	rs5015480	C	EUR [74]	0.58	1.13 (1.07–1.19)	C	EA [120]	0.21	1.17 (1.11–1.24)				
8	rs7903146	T	EUR [99]	0.28	1.65 (1.28–2.02)	T	EA [144]	0.03	1.54 (1.36–1.74)	T	SA [178]	0.28	1.33 (1.19–1.49)
9	rs2237892	C	EA [113]	0.67	1.40 (1.34–1.47)	C	EA [113]	0.93	1.29 (1.11–1.50)	C	HIS [149]	0.79	1.09 (1.06–1.12)
10	rs7178572	G	SA [92]	0.44	1.09 (1.06–1.12)	G	EUR [171]	0.68	1.11 (1.07–1.15)				
11	rs8050136	A	EUR [98]	0.46	1.17 (1.12–1.22)	A	SA [151]	0.25	1.16 (1.09–1.24)				
12	rs4430796	G	EUR [116]	0.51	1.14 (1.08–1.20)	G	EA [146]	0.25	1.19 (1.13–1.25)				
13	rs5945326	A	EUR [116]	0.78	1.27 (1.18–1.37)	A	EA [146]	0.66	1.18 (1.13–1.23)				
14	rs4712523	G	EA [144]	0.41	1.27 (1.21–1.33)	G	EUR [115]	0.34	1.20 (1.14–1.26)				

Population. EUR: European; SA: South Asian; EA: East Asian; HIS: Hispanic.
